# Mesenchymal Stem Cell-Derived Microparticles: A Promising Therapeutic Strategy

**DOI:** 10.3390/ijms150814348

**Published:** 2014-08-18

**Authors:** Xi Tan, Yong-Zhen Gong, Ping Wu, Duan-Fang Liao, Xi-Long Zheng

**Affiliations:** 1 Division of Stem Cell Regulation and Application, College of Medicine, Hunan University of Traditional Chinese Medicine, Changsha 410208, China; E-Mails: xitan5005@gmail.com (X.T.); gongyongzhen@126.com (Y.-Z.G.); pingwu@xtu.edu.cn (P.W.); 2 Department of Biochemistry & Molecular Biology, Libin Cardiovascular Institute of Alberta, Smooth Muscle Research Group, University of Calgary, 3330 Hospital Drive N.W., Calgary, AB T2N4N1, Canada; 3 Department of Pharmacology, School of Pharmaceutical Sciences, Central South University, Changsha 410078, China

**Keywords:** mesenchymal stem cells, microparticles, intercellular communication, clinical therapy

## Abstract

Mesenchymal stem cells (MSCs) are multipotent stem cells that give rise to various cell types of the mesodermal germ layer. Because of their unique ability to home in on injured and cancerous tissues, MSCs are of great potential in regenerative medicine. MSCs also contribute to reparative processes in different pathological conditions, including cardiovascular diseases and cancer. However, many studies have shown that only a small proportion of transplanted MSCs can actually survive and be incorporated into host tissues. The effects of MSCs cannot be fully explained by their number. Recent discoveries suggest that microparticles (MPs) derived from MSCs may be important for the physiological functions of their parent. Though the physiological role of MSC-MPs is currently not well understood, inspiring results indicate that, in tissue repair and anti-cancer therapy, MSC-MPs have similar pro-regenerative and protective properties as their cellular counterparts. Thus, MSC-MPs represent a promising approach that may overcome the obstacles and risks associated with the use of native or engineered MSCs.

## 1. Introduction

In multicellular organisms, homeostasis results from a dynamic balance between cell proliferation and degenerescence [1]. Cells proliferate, differentiate and fulfill particular functions, then undergo programmed apoptosis and are finally cleared by phagocytosis. At each stage of these processes, cells are subjected to a variety of stimulations and release submicron fragments from the cell membrane. Several terms have been used to describe these membrane fragments, such as microparticles (MPs), secretory vesicles, microvesicles, ectosomes or exosomes, depending on the context in which they are studied and what particular properties are investigated [2,3,4,5,6,7,8,9,10,11]. Indeed, a wider consensus as to the nomenclature still needs to be reached in this area [2]. Because different studies use different criteria to describe various MPs, the comparison turns out to be difficult and the nomenclature often becomes inconsistent. In this article, we collectively refer to the vesicles released from mesenchymal stem cells (MSCs) as MSC-MPs, which may be the most commonly used term at the moment, but not necessarily the most precise one.

MSCs derived from adult bone marrow are multipotent and have a limited, but robust potential to differentiate into mesenchymal cell types, such as adipocytes, chondrocytes and osteocytes, with negligible risk of teratoma formation. MSCs present several therapeutically advantageous features, such as easy isolation and acquisition, fast expansion in culture, feasibility of autologous transplantation and their significant paracrine effects. MSCs have various potential therapeutic applications in regenerative medicine and tissue engineering and are currently under clinical trials to treat a wide range of diseases. Notably, recent studies have suggested that the beneficial effects of MSCs do not result from their differentiation, but from the activation of a protective mechanism. This speculation has been supported by the fact that MPs produced from MSCs inhibit apoptosis and fibrosis, potentiate regenerative effects, stimulate mitosis and differentiation of tissue-intrinsic progenitor cells [12] and modulate the immune response [13].

In this review, we have summarized the nature of MSC-MPs and discussed the potential mechanism through which MSC-MPs act on target cells and the experimental evidence suggesting their roles in clinical therapies. The possibility for engineered MSC-MPs to be used as therapeutic agents has also been discussed.

## 2. Characterization of Mesenchymal Stem Cells (MSCs)

MSCs were originally identified by Friedenstein and his colleagues as the primary transplantable components of the bone marrow microenvironment and necessary for the maintenance of definitive hematopoiesis [14]. More specifically, Friedenstein *et al*. defined MSCs as fibroblastic and mesodermally-derived cells that are clonogenic and adherent [14,15,16]. The clonogenic expansion, which was measured in terms of colony forming unit-fibroblast, was presumably the mechanism through which the marrow microenvironment could survive through myeloablative conditioning and facilitate hematopoietic reconstitution. Their original studies revealed several characteristics of MSC, which are still the primary hallmarks.

## 3. Therapeutic Potential of MSCs

### 3.1. Therapeutic Applications of MSCs

MSCs can be isolated from different sources, particularly bone marrow and adipose tissues. They can differentiate into various cell types of the mesodermal germ layer. Two main characteristics of MSCs make them a good candidate for therapeutic applications. At first, they can enter the blood circulation and home in to the sites of damaged and cancerous tissues, where MSCs can release a multitude of trophic factors. In addition, MSCs have the ability to suppress the immune system through various mechanisms. Essentially, both properties of MSCs can be exploited in the field of regenerative medicine and the development of cancer therapies.

#### 3.1.1. MSCs in Tissue Regeneration

The MSCs have various effects on cell functions, such as multi-differentiation, immunosuppression and immune privilege. Unlike embryonic stem cells (ESCs), there is no ethical issue for the use of MSCs. These unique advantages make MSCs a promising cell source for cytotherapy. MSCs have been employed in an increasing number of clinical studies to enhance tissue regeneration following the injuries of both skeletal damage, such as bone [17,18,19], cartilage [20], spinal cord injuries [21,22] and non-skeletal diseases, such as type I diabetes mellitus [23,24], Crohn’s diseases [25,26] and post myocardial infarction [27,28].

#### 3.1.2. MSCs in Tumor Therapy

In this rapidly expanding field, more and more concerns have been raised about the interrelationship between tumor and stem cells. Tumor cells induce the migration of MSCs to the tumor site through secretion of some specific factors [29,30,31,32]. This ability of MSCs to home in to tumor sites has been exploited to develop different types of cancer therapies. Thus, MSCs have been successfully used as the vehicles to efficiently deliver oncolytic viruses into tumors and their metastatic sites in xenograft models of breast carcinoma [33], glioma [34,35] and ovarian cancer [36]. Importantly, the efficiency of this type of therapy has been proven in infant patients with metastatic neuroblastoma refractory to front-line therapies [37]. The ability for MSCs to specifically reside in multiple tumors makes them extremely attractive for directed cancer therapy. However, MSCs are not simple inert vectors, but active cells. It is well known that the presence of cancer stem cells (CSCs) in the main tumor mass may result in classical therapeutical failures [38,39]. Increasing evidence suggests that sarcomas could be good examples of the CSC model, and MSCs might be the target cells for the transforming mutations that give rise to these types of tumors. Thus, several types of human sarcomas have been reproduced *in vivo* through the over-expression of specific fusion oncoproteins in MSCs [40,41]. In addition, CSCs displaying MSC properties have been recently identified in Ewing’s sarcoma [42]. Taken together, these findings suggest that MSCs may contribute to the tumor microenvironment and can be exploited as the target cell of therapy for tumors with mesenchymal origin (especially sarcomas). In conclusion, MSCs can be a foe or friend of cancer. More detailed studies about the roles of MSCs in cancer progression will undoubtedly lead to a safer and more effective clinical anticancer therapy in the future.

### 3.2. Limitations of MSC Therapy

The use of MSCs to treat human diseases has raised several concerns in the past decade. There have been several challenges identified in this area. The challenges related to transplanted MSCs include senescence-induced genetic instability, immune-mediated rejection or loss of function and limited cell survival [43]. The major problem in the clinical applications of MSCs is their potential malignant transformation. The production of a sufficient number of MSCs for clinical use requires a consistent *in vitro* expansion, which may lead to their spontaneous transformation [44]. Furthermore, genetic manipulations of MSCs for the treatment of different diseases can *per se* increase the oncogenic potential of the cells, because the transgenes may be tumorigenic and/or cause disruptions in the genome. MSCs have been found in a number of tumors, including gastric adenocarcinoma [45], lipoma [46] and osteosarcoma [47], strongly suggesting their involvement in tumor development. In addition, various studies indicate that MSCs are the potential sources of tumor-associated fibroblasts [48]. For these reasons, any application of MSCs in the clinical setting should be cautiously evaluated.

Compared with cell therapy with MSCs, the therapeutical application of MPs has more advantages. MSC-MPs, for example, are more preservable and stable [49]. They induce stronger signaling [50], and their functions do not exhaust over time [51]. Moreover, MSC-MPs have no risk of aneuploidy [52,53], immune rejection or teratoma formation after *in vivo* allogeneic administration [54]. Hence, the idea of mass produced “universal donor” stem cells for the treatment of diseases will be a promising therapeutic strategy through the application of MSC-MPs as an accessory therapeutic derivation of MSCs.

## 4. Characterization of MSC-MPs

MPs are the fragments of cell membranes released by stimulated or apoptotic cells, such as stem cells, endothelial cells, erythrocytes, platelets, monocytes and lymphocytes, ranging in size from 0.1 to 1 μm, and are yet to be clearly defined. A number of triggers, which induce cell activation, apoptosis and MP formation, have been identified, including cytokines (such as tumor-necrosis factor, interleukin-6 (IL-6)), thrombin and endotoxin, shear stress and hypoxia [55,56]. To explore the underlying mechanism of MSC-MP formation, for example, apoptosis of rat bone marrow MSC was induced by either hypoxia or serum-free starvation, followed by the analysis of the subcellular structures in the supernatants. The results showed that MSC could release MPs in response to hypoxia or culture with serum-free medium, and the amount of these membrane MPs was around a 15-fold increase compared with those in unstimulated cells. This study provides some novel information about the mechanisms underlying the formation of MSC-MPs [57]. Two distinct mechanisms involving membrane remodeling and cytoskeleton disruption have been proposed for the production and shedding of the majority of cell derived-MPs, which helps us better understand the MSC-MP formation [58,59,60,61,62,63,64,65,66,67,68]. Although the exact mechanism remains to be illustrated, MSC-MPs have become a novel potential therapeutic tool.

**Figure 1:**
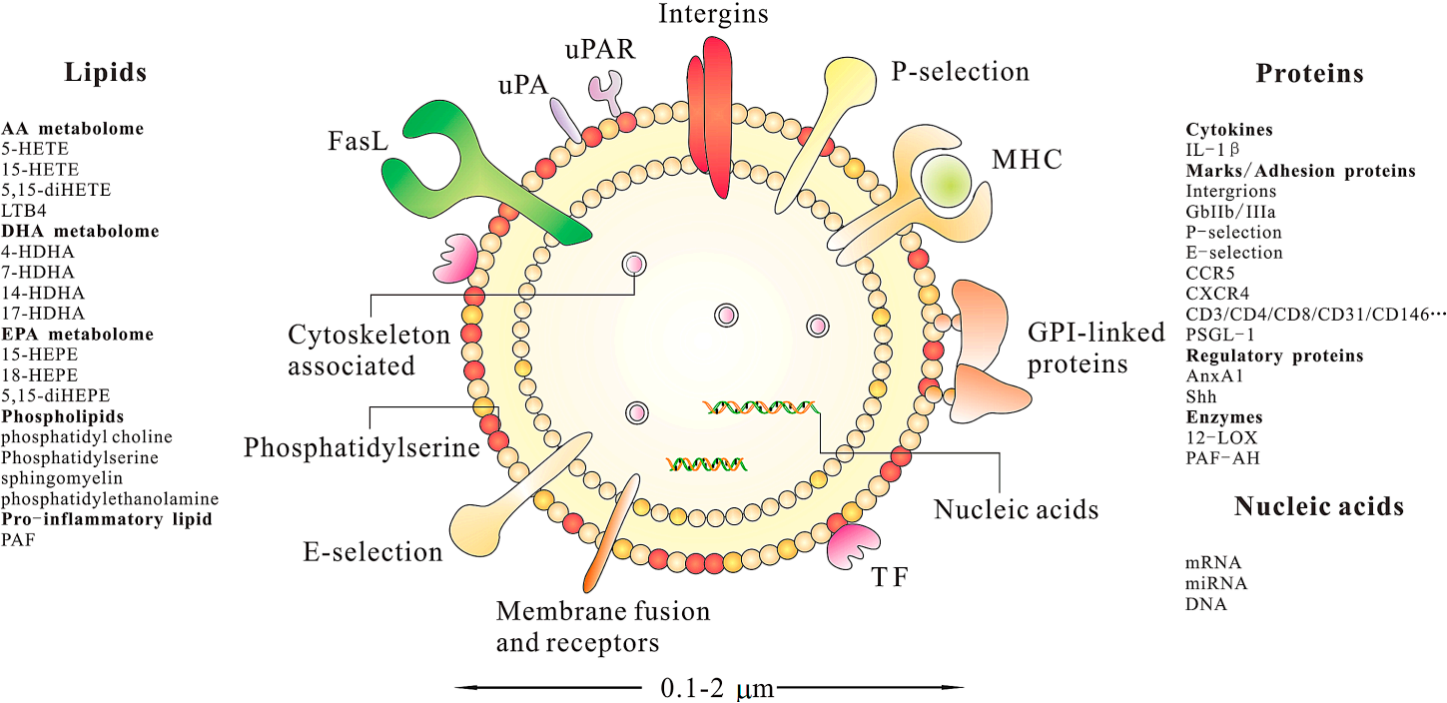
Schematic illustration of a cell-derived microparticle (MP). These biomolecules are identified from diverse research publications. This presentation does not intend to be exhaustive with respect to the differentcomponents of MPs. IL-1β, interleukin-1β; GPI, glycosylphosphatidylinositol; GPIIb/IIIa, glycoprotein IIb/IIIa; CCR5, C–C chemokine receptor type 5; CXCR4, C–X–C chemokine receptor type 4; PSGL-1, P-selectin glycoprotein ligand-1; AnxA1, Annexin A1; Shh, Sonic Hedgehog; 12-LOX, 12-lipoxygenase; PAF-AH, platelet-activating factor acetylhydrolase; HETE, hydroxy-eicosatetraenoic acid; LTB4, leukotriene B4; HDHA, hydroxy-docosahexaenoic acid; HEPE, hydroxyeicosapentaenoic acid; PAF, platelet activating factor.

MSC-MPs, the secreted bi-lipid membrane vesicles of endosomal origin [69], contain a wide range of biomolecules: proteins (signal transduction and effector proteins, receptors, cytoskeleton), lipids and even nucleic acids (mRNA, microRNA or miRNA and DNA) [70]. The protein and lipid components of MSC-MPs are important parameters to determine their biological effects. As a bi-lipid membrane vesicle, MSC-MPs have the capacity not only to carry a large cargo load, but also to protect the contents from chemicals or degradative enzymes. The protein and RNA in MSC-MPs, for example, can be protected from degradation by trypsin and RNase, as long as the lipid membrane is not compromised [49,71]. Lai *et al*. also found that storage of MSC-MPs without the addition of potentially toxic cryopreservatives at −20 °C for six months did not compromise their protective effects or their biochemical activities [69]. Figure 1 summarizes the composition and potential properties of a canonical MP. Despite the interest raised by MSC-MPs for their potential roles in physiological and pathological conditions and their possible applications in the treatment of various diseases, only a few studies have been conducted on the nucleic acids and protein contents of MSC-MPs.

### 4.1. Nucleic Acids

Several studies have revealed the presence of different types of nucleic acids in MSC-MPs, suggesting that MSC-MPs could transfer genetic materials to other target cells [71,72,73]. The presence of some specific miRNAs has been reported within MSC-MPs by Collino *et al*. [72]. In these studies, a comparative miRNA profiling was performed with arrays using bone marrow and tissue-specific MSCs and their respective MPs. The authors found that some miRNAs were present in both MPs and their original cells. It is interesting to note that some miRNAs are selectively sorted into the MPs, which were undetectable in the cells. However, some other miRNAs are present only in the cells and not in the MPs. These observations suggest a specific mechanism controlling the sorting of miRNAs in MSC-MPs. Moreover, MSC-MPs have been shown to contain precursor miRNAs, as well [71], and their abnormal expression seems to be associated with the development of cardiovascular diseases and cancer [74,75]. In addition, MPs contain mRNA for the proteins, such as the receptors of specific growth factors. For instance, MPs released by bone marrow MSCs contain mRNA for the insulin growth factor 1 (IGF-1) receptor [73]. In an *in vitro* model of renal toxic injury induced by cisplatin, the transfer of the IGF-1 receptor mRNA through MPs has been shown to increase the proliferation of damaged proximal tubular cells [73]. Taken together, all of this evidence implies that MSC-MPs may act as the vectors of genetic-related messages between cells.

### 4.2. Proteins

Besides the nucleic acids in MSC-MPs, the proteome is equally important in terms of the functions of MSC-MPs. However, only one study has been conducted so far to characterize the protein composition of MSC-MPs in detail. By characterizing the contents of bone marrow MSC-MPs, Kim *et al.* [76] identified 730 MP proteins, among which there were some mediators controlling self-renewal and differentiation. Interestingly, their analysis revealed a number of surface markers, such as the epidermal growth factor (EGF) receptor, platelet-derived growth factor (PDGF) receptor-B and the plasminogen activator urokinase receptor (PLAUR), signaling molecules of the RAS-MAPK (RRAS/NRAS, MAPK1 and VAV2), RHO (GNA13) and CDC42 (GNG12 and CDC42) pathways. In addition, they include cell adhesion molecules and additional MSC antigens that support a possible role for MSC-MPs in tissue regeneration through their effects on MSC migration [76]. According to these findings, MSC-MPs appear to hold many characteristics of the MSCs themselves and may be important for the functions of these adult stem cells *in vivo*.

### 4.3. Lipids

The structure analysis has revealed that MP is surrounded by a phospholipid bilayer [77]. The phospholipid composition of MPs from healthy people consists mainly of phosphatidyl choline (approximately 60%), with the remainder composed of sphingomyelin, phosphatidylethanolamine and phosphatidylserine [78]. Notably, externalization of phosphatidylserine provides an efficient platform for the assembly of blood coagulation enzymes, leading to the activation of the coagulation cascade [79].

## 5. Therapeutic Potential of MSC-MPs

### 5.1. Intercellular Communication and Phenotypic Change

**Figure 2:**
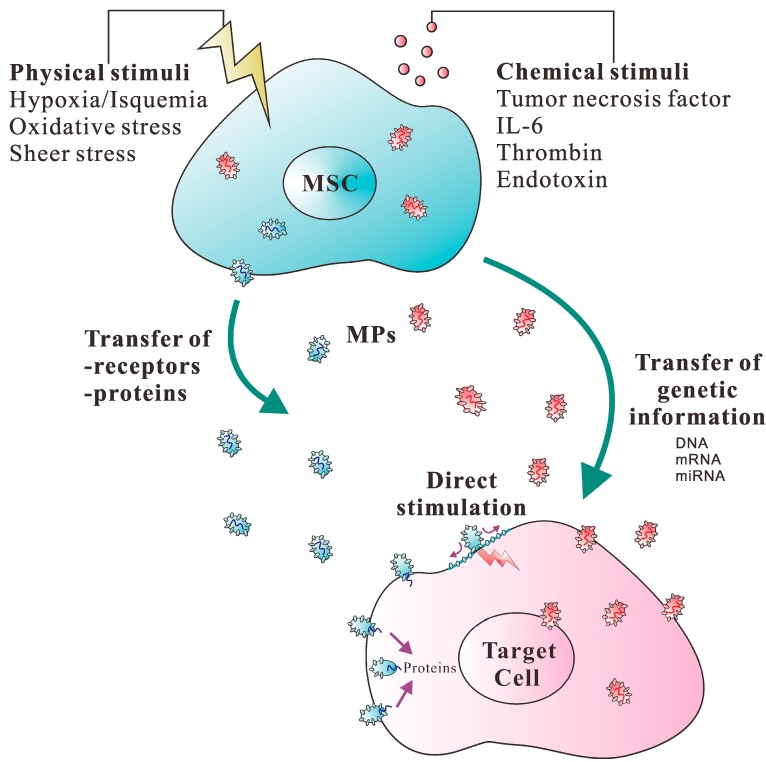
Schematic presentation of the mechanisms involved in MP-mediated intercellular communication. After MSCs are activated by physical and/or chemical stimuli, MPs are produced through the disruption of cytoskeleton. (a) MPs may transfer receptors or other proteins from the cell of origin to the target cell; (b) MPs may directly stimulate target cells through their cell membrane receptors; (c) MPs may convey genetic information by horizontal transfer of DNA, mRNA, miRNA or transcription factors to induce functional changes in the target cell.

Cells communicate and exchange information by several different methods, including the secretion of soluble factors, the intercellular exchange of organelles and cell-to-cell adhesion contact through nanotubular structures [80]. Recent studies have proposed that MPs, the cell-derived small circular membrane vesicles, represent an additional mechanism underlying cell-to-cell communication [81,82], since these vesicles have the ability to transfer proteins and functional genetic material, such as RNA, to other cells, as discussed earlier [83,84,85,86]. It is therefore possible that MSCs also transfer bio-active lipids, proteins and nucleic acids to the target cells via MPs. Supportively, the transfer of MSC-MPs induces phenotypic and functional changes in the recipient cells that promote the activation of regenerative programs. For instance, MSCs release a significant amount of MPs containing mRNA with multiple specific differentiative and functional properties, as well as selected patterns of mature miRNAs [54,72]. These nucleic acids can be transferred via MPs to the target cells to induce their functional and phenotypic changes. This observation engenders the possibility that stem cells may exert their biological effects by delivering genetic information and altering the gene expression of target cells through the MP-mediated transfer of mRNA and miRNA. In addition, it has been demonstrated that MPs released from human MSCs and liver stem cells also contain ribonucleoproteins involved in the intracellular trafficking of RNA, suggesting that it is possible to dynamically regulate and compartmentalize RNAs by MPs derived from human adult stem cells of mesenchymal origin [72]. The mechanisms underlying MP-mediated cell-cell communication have been proposed in Figure 2.

### 5.2. Therapeutic Applications of MSC-MPs

MPs derived from MSCs could represent a novel potential therapeutic tool. Given that MPs deliver bioactive cargo and exert significant effects on the target cells, the use of MPs as a treatment approach has been exploited in several diseases, such as cardiovascular diseases, renal diseases and cancer.

#### 5.2.1. Cardiovascular Disease

The potential use of MSC-MPs for the treatment of cardiovascular diseases has recently been reported by Lai *et al*. [69]. In a mouse model of myocardial ischemia-reperfusion, the authors previously demonstrated the therapeutic activity of MPs isolated from the ESC-derived MSCs [49]. They concluded that the secretion of protective MPs is a general property and perhaps a predominant function of MSCs and probably related to the supporting role of the stromal cells. Considering the limitations and costs related to the use of ESC and the large number of cells required for MP production, the same authors also generated Myc-immortalized ESC-derived MSCs and demonstrated that MPs derived from these cells still display their original cardioprotective activity [87]. Those results suggest that MSC-MPs may be developed as a therapeutic tool for cardioprotection in the near future.

#### 5.2.2. Renal Disease

There have been many applications of MSCs in the treatment of kidney diseases, in which MSCs could prevent the process of deterioration or reduce injury following toxic or ischemic events or affect kidney diseases through other repair processes [73,88,89,90,91]. Recently, it has been demonstrated that MPs released from MSCs favor renal repair in non-lethal toxic and ischemic acute kidney injury (AKI). MSC-MPs may be a paracrine mechanism for cell-to-cell communication with reversing function after kidney injury. For example, MPs derived from human MSCs stimulated proliferation and inhibited the apoptosis of tubular epithelial cells *in vitro* and were found to be as effective as MSCs in accelerating functional and morphological recovery from glycerol-induced AKI in immunodeficient mice [72]. A recent study has also examined the effect of MSC-MPs in a model of kidney injury induced by ischemia-reperfusion injury (IRI) in immunocompetent rats [72]. It showed that a single administration of MPs derived from adult MSCs immediately after the induction of IRI protects rats against AKI and chronic kidney disease development [92]. In the *in vivo* model, MPs seem to exert renal protection by limiting the extent of injury due to their significant reduction of tubular cell apoptosis and significant stimulation of the proliferation and survival of tubular cells. These effects of MPs suggest that they limit the initial renal injury and may also protect the kidney from the development of chronic injury. In conclusion, MPs released from MSCs may mimic the effects of the original cells, suggesting that MSC-MPs could be exploited as a new therapeutic approach for renal regeneration.

#### 5.2.3. Cancer

Although evidence has been provided to support the protective and beneficial roles of MSC-MPs in tissue repair, it has been reported that MSC-MPs have dual effects on tumor growth: promoting angiogenesis and tumor initiation and inhibiting the progression of established tumors. Recent studies demonstrated that MPs are an integral component of intercellular communication within the tumor microenvironment. Investigators found that MSC-MPs inhibited cell cycle progression and induced the apoptosis of HepG2 and Kaposi’s cells, as well as the necrosis of Skov-3 cells. They demonstrated that MSC-MPs induce the *in vitro* cell cycle arrest, apoptosis or necrosis of different tumor cell lines and inhibit the growth of established tumors *in vivo* [93]. MP administration may have some advantages with respect to MSCs, because MPs inhibit the cell cycle progression of tumor cells without the risk of MSC differentiation into stromal fibroblasts that may favor tumor growth [94,95]. Instead, bone marrow MSC-MPs have been shown to support tumor growth and angiogenesis in a mouse xenograft model of gastric carcinoma, and the pro-angiogenic effect has been ascribed to the increase of vascular endothelial growth factor (VEGF) expression in cancer cells [96]. This finding is not completely unexpected, since MSCs have been reported to have various tumor-promoting effects [94]. This highlights once more that we must be cautious when evaluating the risks related to the use of MSCs or MSC-MPs in anticancer therapy.

## 6. Engineered MSC-MPs for Therapy

Although MSC-MPs have both positive and negative effects on diseases, profound therapeutic effects have been observed for felicitously engineered MSC-MPs. In order to exploit MSC-MPs as therapeutic agents, it is necessary to produce them on a large scale. There are several possible ways to produce and modify MSC-MP composition. At first, MPs for applications in clinical therapies can be isolated from MSCs. It is possible to establish MP-producing cell lines for this purpose; Secondly, MP-producing cell lines could be transduced with special genes to over-express: (1) proteins that prevent target cells in injured tissues from apoptosis and that stimulate the proliferation of residual cell populations (Notch ligands or stem cell factor); or (2) factors that effectively induce angiogenesis (vascular endothelial growth factor, stromal cell-derived factor 1 or fibroblast growth factor 2); Thirdly, it is our speculation that MPs derived from cells in hypoxic conditions will be rich in mRNAs and miRNAs to promote angiogenesis. Furthermore, MP-producing cell lines can be rich in mRNA and miRNA species, which will promote regeneration after delivery to the injured tissues; Finally, we speculate that MP-producing cell lines could be rich in components to help their tropism better match the injured organ and prolong MP retention in the injured tissues. In summary, the release of MPs from founder cells can be enhanced by proper manipulation of culture conditions, and MPs can be custom-engineered to make them more suitable for therapy.

## 7. Prospects and Conclusions

MPs, once thought of merely as cell debris, now seem to influence a diverse series of physiological (such as cell communication and stem cell plasticity) and pathological processes (such as the spread of disease and the repair of injured tissues). Given that MPs mimic MSC properties, MSC-MPs might be developed to improve current clinical trial outcomes that use adult cells. While the use of MSC-MPs in regenerative medicine and anti-cancer treatment has a promising future, further investigation is required in several areas: (1) the stimuli and the molecular pathways that regulate the assembly of the biologically active molecules within MSC-MPs; (2) the potential application of MSC-MPs as diagnostic tools in different pathological conditions; (3) the strategies to reduce MP formation and/or to remove unwanted MPs from circulation; and (4) the exploitation of the therapeutic potential of MSC-MPs through modifying the phenotype and function of target cells.

In conclusion, to further explore the potential clinical use of MSC-MPs, much more studies and multidisciplinary investigations (biology, regenerative medicine and engineering) are needed to better understand these emerging concepts.
